# Clinical Profile and Visual Outcomes of Ocular Syphilis: A Five-year Review in Hospital Universiti Sains, Malaysia

**DOI:** 10.7759/cureus.4015

**Published:** 2019-02-05

**Authors:** Ahmad Kamal Ghanimi Zamli, Noor Shirlyna Irma Ngah, Tan Chew-Ean, Julieana Muhammed, Wan-Hazabbah Wan Hitam, Adil Hussein, Embong Zunaina

**Affiliations:** 1 Ophthalmology, Universiti Sains Malaysia, Kota Bharu, MYS

**Keywords:** ocular syphilis, uveitis, neurosyphilis, human immunodeficiency virus (hiv) negative

## Abstract

Introduction

Ocular syphilis is a sight-threatening condition. It can occur at any stage of syphilis infection, which present either with acute inflammation during the primary, secondary, and early latent stages or with chronic inflammation during tertiary infection, affecting virtually every ocular structure. This study was to report on the clinical presentation of ocular syphilis that presented to eye clinic Hospital Universiti Sains Malaysia.

Methodology

This was a retrospective study where medical records of ocular syphilis patients who attended eye clinic in Hospital Universiti Sains Malaysia from January 2013 to June 2017 were reviewed.

Results

A total of 10 patients (13 eyes) with ocular syphilis were identified out of 106 cases that presented with ocular inflammation. The mean age of presentation was 69.8 ± 6.4 years and seven of them (70%) were female. All patients were Malay and human immunodeficiency virus (HIV) was negative. The ocular manifestations included panuveitis (four eyes, 30.8%), anterior uveitis (two eyes, 15.4%), posterior uveitis (seven eyes, 53.8%) and optic neuritis (two eyes, 15.4%). Seven (53.8%) eyes presented with visual acuity of worse than 6/60, five (38.5%) eyes had visual acuity between 6/15 to 6/60, and one (7.7%) eye had visual acuity of 6/12 or better. Nine patients received an intravenous benzylpenicillin regime and one patient received an intramuscular penicillin injection. Out of 13 eyes affected, 11 (84.6%) eyes had improved visual acuity of at least one Snellen line after treatment. Visual acuity of 6/12 or better increased to four (30.8%) eyes.

Conclusions

Posterior uveitis was the commonest presentation of ocular syphilis in HIV-negative patients. Early detection and treatment of ocular syphilis can result in resolution of inflammation and improvement of vision.

## Introduction

Ocular syphilis is an uncommon presentation of *Treponema pallidum* infection. Since the advent of antibiotics, the rate of ocular syphilis infection has decreased significantly, thus reducing the complications that arise from chronic syphilitic infection. In 1950, ocular syphilis was among the major causes of blindness, making up an estimated 10% to 15% of cases resulting in total blindness in the United States (US) [1]. The prevalence of ocular syphilis has decreased dramatically to 0.6% of total syphilis cases in the year 2000 and onwards [2]. However, recently, prevalence is increasing dramatically due to the worldwide human immunodeficiency virus (HIV) infection epidemic.

Diagnosing ocular syphilis proves to be challenging. This occurs as a result of its diverse clinical manifestations [3]. In fact, due to the rarity of the eye infection due to syphilis, ocular syphilis can be underdiagnosed and overlooked by a primary physician treating the syphilis infection [4]. All of these factors contribute to its debilitating visual sequelae and impair the chances of effective immediate treatment.

Ocular syphilis, which can manifest at any stage of the syphilis infection, occurs more frequently during the latent stage of syphilis infection than during the primary stage [4-5]. In immunocompetent patients, ocular syphilis presents more often in the elderly, while in younger patients, it is usually a result of HIV coinfection [6]. Visual prognosis is strongly associated with the anatomical structure that is affected by the syphilis infection, and it implies poor prognosis when it involves the macula [7]. 

The most common ocular syphilis presentation is uveitis [8]. However, the frequency and location of uveitis (anterior, intermediate, posterior, or panuveitis) vary greatly between each study reviewed [9-11]. Other rarer presentations of ocular syphilis, such as stromal keratitis, iridocyclitis, necrotizing retinitis, optic atrophy, and retinal vasculitis, are only found in patients that have long-standing tertiary syphilis [8]. This retrospective study aims to report on demographic data, various clinical manifestations, treatments, and visual outcomes of ocular syphilis in a tertiary eye centre in Malaysia (Hospital Universiti Sains Malaysia).

## Materials and methods

We conducted a retrospective study to review the clinical profiles and visual outcomes of 10 patients (presenting symptoms in 13 eyes) with ocular syphilis treated at the Hospital Universiti Sains Malaysia from January 2013 to June 2017. The follow-up period ranged from two months to 48 months.

We selected patients diagnosed with ocular syphilis based on history, clinical evidence of ocular inflammation not attributable to any other cause, and positive serology for *Treponema pallidum*. Serological tests include rapid plasma reagin (RPR), fluorescent treponemal antibody absorption (FTA-ABS), *T. pallidum* particle agglutination (TP-PA), and electrochemiluminescence immunoassay (ECLIA) for syphilis. Patients presumed to have ocular syphilis were also investigated to exclude other possible causes of ocular infection, such as tuberculosis, toxoplasmosis, herpes simplex virus (HSV), and cytomegalovirus (CMV) and HIV. Cerebrospinal fluid (CSF) analysis was requested from our patients but none of them consented for the procedure.

Data were collected on demographic features (age, sex, and race), history of high-risk sexual behavior, past medical history, past history of syphilis infection, onset and duration of ocular symptoms at presentation, visual acuity, anterior and posterior segment examination, treatment regime, and visual outcome. Visual outcome was evaluated based on best-corrected visual acuity at the latest follow-up ranged from two months to 48 months after completion of treatment. Good visual outcome is defined as visual acuity of 6/12 or better, while a poor outcome constitutes a visual acuity below 6/60.

The data were analyzed using the Statistical Package for Social Science, version 22.0 (SPSS Inc., Chicago, IL, USA). This study was performed in accordance with the ethical standards of The Human Research Ethics Committee of Universiti Sains Malaysia (reference number: USM/JEPeM/19010009) and was in line with the Declaration of Helsinki of 1975, as revised in the year 2000.

## Results

A total of 10 patients (13 eyes) with ocular syphilis were identified within the study period (2013-2017). These cases comprised 9.4% of 106 new cases that presented with uveitis in our centre during the specified time period. Seven (70.0%) patients were female, and the mean age of presentation was 69.8 ± 6.4 years (range, 60-80 years). All of them were from the Malay ethnic group. One patient had a history of multiple sexual partners, while the rest denied any high-risk sexual behavior or intravenous drug abuse. All patients were heterosexual and denied any homosexual activity.

Three (30.0%) patients had both eyes involvement. The mean duration of ocular symptoms at the time of initial visit was 11.4 ± 16.7 months (ranged from one week to four years). At the time of presentation, blurring of vision was the commonest presenting complaint (92.3%, 12 eyes), followed by redness of eye (23.1%, three eyes) and eye pain (7.7%, one eye). Seven (53.8%) eyes presented with visual acuity of worse than 6/60, while five (38.5%) eyes had visual acuity between 6/15 to 6/60. There was only one (7.7%) eye with visual acuity of 6/12 or better.

Vitritis was the commonest finding during ocular examination and was found in five eyes (38.5%). This finding was followed by keratic precipitates (30.8%, four eyes), anterior chamber cells (30.8%, four eyes), and chorioretinitis (30.8%, four eyes). Other ocular findings included macular oedema (23.1%, three eyes), retinal scar or atrophy (23.1%, three eyes), posterior synechiae (15.4%, two eyes), optic disc swelling (15.4%, two eyes), ectropion uvea (7.7%, one eye), Roth’s spot (7.7%, one eye), vitreous hemorrhage (7.7%, one eye) and retinal hemorrhages (7.7%, one eye).

Out of these 13 eyes involved, a large proportion of the eyes (53.8%, seven eyes) had posterior uveitis. Four (30.8%) eyes had panuveitis and two (15.4%) eyes had anterior uveitis, while another two (15.4%) eyes had optic neuritis. Three patients with bilateral eye involvement were noted to have posterior uveitis. The above information is presented in Table 1.

**Table 1:** Clinical profile of patients with ocular syphilis RE, right eye; LE left eye; BE, both eyes; BOV, blurring of vision; HPT, hypertension; IHD, ischemic heart disease; CKD, chronic kidney disease; CVA, cerebrovascular accident; N, normal; KP, keratic precipitates; BK, bullous keratopathy; SP, seclusio pupillae; PS, posterior synechia; IOP, intraocular pressure; mmHg, millimetres of mercury; CDR, cup-disc-ratio; PACG, primary angle closure glaucoma; AC, anterior chamber; VH, vitreous haemorrhage; DH, dot haemorrhage; BH blot haemorrhage; OD, optic disc; IV, intravenous; IM, intramuscular; MU, megaunit; BCVA, best corrected visual acuity; NPL, no perception of light; CF, counting fingers; HM, hand movement; ft, feet

No	Age	Sex	Eye	Ocular symptoms	Duration (months)	High-risk behaviour	Comorbid	Ocular examination		Clinical diagnosis	Treatment	Initial BCVA		Final BCVA		Follow-up duration (months)
								RE	LE			RE	LE	RE	LE	
1	61	F	RE	BOV	1 week	Husband has syphilis	Nil	KP, AC cells	N	RE anterior uveitis	IM penicillin 2.4 MU weekly for 3 weeks	6/24	6/7.5	6/7.5	6/7.5	48
2	69	F	RE	RE BOV	36	Unknown	Nil	KP, SP, AC cells, chorioretinitis, macular edema, IOP 12 mmHg, CDR 0.9	BK, IOP 26mmHg	RE panuveitis, BE PACG	IV benzylpenicillin 4 MU 4 hourly for 2 weeks	6/36	NPL	6/15	NPL	40
3	67	F	BE	BOV, Eye redness, Eye pain	2	Unknown	Nil	Chorioretinitis, VH	Chorioretinitis, Chorioretinal scar	BE posterior uveitis	IV benzylpenicillin 3 MU 4 hourly for 2 weeks	6/45	6/12	HM	6/9	40
4	71	F	BE	BOV	3	Unknown	DM, HPT	Chorioretinitis, Macular scar	Roth’s spots, Vitritis, DH, BH	BE posterior uveitis	IV benzylpenicillin 4 MU 4 hourly for 2 weeks	CF 2ft	CF 2ft	6/90	6/24	40
5	67	M	LE	BOV	3	Unknown	Nil	N	AC cells, vitritis, macular edema	LE panuveitis	IV benzylpenicillin 4 MU 4 hourly for 2 weeks	6/9	CF 2ft	6/9	4/60	30
6	80	F	BE	BOV	3	Unknown	HPT, IHD CKD, CVA	OD swelling, macular edema	OD swelling, peripheral chorioretinal atrophy	BE posterior uveitis, BE optic neuritis	IV benzylpenicillin 3 MU 4 hourly for 2 weeks	3/60	6/60	6/21	6/7.5	12
7	76	F	LE	Recurrent eye redness	48	Unknown	Nil	Senile cataract	PS, AC cells, ectropion uvea, senile cataract	LE anterior uveitis, RE cataract	IV benzylpenicillin 4 MU 4 hourly for 2 weeks	1/60	CF 2ft	6/7.5 (Post cataract surgery)	CF 2ft	12
8	75	M	RE	BOV	12	Unknown	HPT, IHD	Vitritis	N	RE posterior uveitis	IV benzylpenicillin 3 MU 4 hourly for 2 weeks	CF 2ft	6/15	6/24	6/15	4
9	60	F	RE	BOV	3 weeks	Sexual promiscuity	HPT	KP, PS, vitritis	N	RE panuveitis	IV benzylpenicillin 4 MU 4 hourly for 2 weeks	6/60	6/9	6/36	6/9	3
10	72	M	LE	BOV	6	Unknown	DM, HPT, thyroid adenoma	N	KP, vitritis	LE panuveitis	IV benzylpenicillin 4 MU 4 hourly for 2 weeks	6/6	6/90	6/6	6/12	2

All patients underwent both treponemal (either ECLIA, TP-PA or FTA-ABS) and non-treponemal tests (RPR) to confirm the diagnosis. Eight patients (80.0%) were tested with ECLIA. Out of these eight patients, six (60.0%) of them were reactive for ECLIA and RPR, one patient (case 2) had positive ECLIA but negative RPR and one patient (case 3) was tested negative for both ECLIA and RPR. In case 2, another treponemal test (TP-PA) was done in order to confirm the diagnosis of ocular syphilis. While in case 3, a repeat treponemal test (FTA-ABS) was done in view of clinical suspicion and was positive for syphilis. In cases 6 and 9, a different treponemal test was done for each patient (FTA-ABS for case 6 and TP-PA for case 9). Both patients tested positive for treponemal and RPR test. From the laboratory data, there was no statistical difference in the titer level of RPR and ECLIA with regard to the severity of visual acuity impairment. HIV testing in all patients was negative. Other blood investigations (serologic testing for toxoplasmosis, CMV, and HSV) were unremarkable. The relevant data are illustrated in Table 2.

**Table 2:** Serologic testing for syphilis and HIV status of patients with ocular syphilis HIV, human immunodeficiency virus; ECLIA, electro-chemiluminescence immunoassays for syphilis; TP-PA, *Treponema pallidum* particle agglutination test; FTA-ABS, fluorescent treponemal antibody absorption test; RPR, rapid plasma reagin test; NR, non-reactive; R, reactive; NT, not tested

Case	Syphilis ECLIA Titre	Syphilis TP-PA	FTA-ABS	Syphilis RPR	HIV status
1	160	NT	NT	1/16	Negative
2	65	Positive	NT	NR	Negative
3	NR	NT	R	NR	Negative
4	283	NT	NT	1/4	Negative
5	86	NT	NT	1/4	Negative
6	NT	NT	R	1/2	Negative
7	66	NT	NT	1/4	Negative
8	103	NT	NT	1/8	Negative
9	NT	Positive	NT	1/2	Negative
10	68	Positive	NT	1/2	Negative

Treatment of ocular syphilis was given in all patients. Nine patients were treated with intravenous (IV) benzylpenicillin 3.0 to 4.0 mega units (MU) for 14 days. One patient was given intramuscular (IM) penicillin 2.4 MU weekly for three weeks (case 1). However, this patient presented to us back again one year later with recurrence of ocular syphilis. She was then treated with IV benzylpenicillin regime and since then had no recurrence within three years' post-treatment. In addition, case 4 also had a recurrence after one year of treatment and was treated successfully with similar IV benzylpenicillin regime.

Out of these 13 eyes, clinical improvement of visual acuity of at least one Snellen line was seen in 11 (84.6%) eyes. Eight (61.5%) eyes had an improvement of at least three Snellen lines. One eye had visual acuity deterioration post-treatment and another one eye had a static visual acuity post IV benzylpenicillin treatment, which was due to the presence of a Senile cataract. Patients with posterior uveitis had visual acuity improvement in six out of seven eyes, of which four (57.1%) eyes had an improvement of three or more Snellen lines. The result of visual acuity of worse than 6/60 reduced from seven (53.8%) eyes to four (30.8%) eyes after completion of treatment. Four (30.8%) eyes had visual acuity of 6/12 or better and five (38.5%) eyes had visual acuity between 6/15 to 6/60.

## Discussion

Syphilis is an ancient disease that still lingers in our society. According to the Malaysian Annual Health report, the rate of syphilis infection has increased from 3.0 cases per 100,000 people in 2010 to 8.0 cases per 100,000 people in 2017 [12-13]. A similar pattern has been observed worldwide. In the US, the rate of primary and secondary syphilis infections has steadily increased from 2.1 cases per 100,000 people in 2000 to 9.5 cases per 100,000 people in 2017 [14]. World Health Organization (WHO) estimates that, as one of the four most common sexually transmitted diseases (followed by chlamydia, gonorrhea, and trichomoniasis), syphilis infection morbidity rates will continue to burden the healthcare system, alongside the increasing number of patients contracting HIV [15].

Ocular syphilis is a manifestation of the chronic syphilis infection. Our study showed that ocular syphilis occurs during the latent stage of syphilis. All patients were also noted to be older than 50, with a mean age of 69.8 ± 6.4. A retrospective study conducted in Korea of ocular syphilis in HIV-negative patients shows similar findings [5]. Shen et al. found the median age of ocular syphilis presentation to be 50.3 ± 5.9 years in their study, which is comparable to the results of our study even though their study included one HIV patient [16]. A late presentation of ocular syphilis can be attributable to the reduced immunity of elderly patients and the possible presence of multiple other comorbidities. Some patients may have even been asymptomatic after exposure to syphilis and therefore left untreated for an extended period of time, allowing disease progression [4].

Most ocular syphilis studies that include both HIV-positive and HIV-negative populations showed a substantial increase of ocular syphilis infection in male individuals. Table 3 summarizes published studies on ocular syphilis from Turkey, Korea, US, China, Singapore, and our current review. A study by Oliver et al. conducted in eight US states reported a 14-fold increase of ocular syphilis infection in male cases (362) compared to female cases (26), regardless of HIV status [2]. Similar observations of gender discrepancies were also noted by Moradi et al., who saw a seven-fold increase in their study conducted at Johns Hopkins Hospital, and by Yap et al. in Singapore, who noted a 10-fold increase in male patients compared to female patients [4,17]. Increased ocular syphilis cases in male were postulated to be attributed to the increased incidence of male patients with HIV and the practice of homosexuality [2,4,6]. This gender discrepancy finding was rarely exhibited in populations of HIV-negative patients where the rate of ocular syphilis was equal amongst males and females [4-5,7]. In our study, ocular syphilis was actually more common in female (70.0%). Factors that contribute to the high frequency of ocular syphilis amongst female patients in our center cannot be obtained because only two patients have known risk factors. This type of response is not uncommon amongst the Malay Muslim society due to the perception of such information being considered taboo and shameful.

**Table 3:** Comparison of current review with previous studies of ocular syphilis N, number of patients; IV, intravenous; IM, intramuscular; HIV, human immunodeficiency virus; +ve, positive; -ve, negative; U, unavailable data; NS, not specified

Variables	Current review	Sahin et al. [7]	Kim et al. [5]	Moradi et al. [4]	Shen et al. [16]	Oliver et al. [2]	Yap et al. [17]
Country/year	Malaysia/2017	Turkey/2016	Korea/2016	US/2014	China/2015	US/2016	Singapore/2014
Duration (total in years)	2013–2017 (5)	2012–2014 (3)	2002–2014 (12)	1984–2014 (31)	2009–2014 (6)	2014–2015 (2)	2004–2009 (6)
N (number of eyes)	10 (13)	12 (17)	39 (45)	35 (61)	13 (21)	388 (U)	12 (18)
Population HIV +ve/-ve	HIV -ve	HIV -ve	HIV -ve	HIV +ve and HIV -ve	HIV +ve and HIV-ve	HIV +ve and HIV -ve	HIV +ve and HIV -ve
HIV +ve	0	0	0	19	1	198	3
HIV -ve	10	12	39	16	12	190	7
Unknown	0	0	0	0	0	0	2
Mean/median age	69.8 ± 6.4	43.8	61.0 ± 11.4	49.4 ± 16.5	50.3 ± 5.9	44.0	49.5
Gender (%)							
Male	3 (30.0)	7 (58.3)	21 (53.8)	26 (74.3)	7 (53.8)	362 (93.3)	11 (91.7)
Female	7 (70.0)	5 (41.7)	18 (46.2)	9 (25.7)	6 (42.9)	26 (6.7)	1 (8.3)
Symptoms (%)							
Blurring of vision	12 (85.7)	U	U	17 (27.9)	11(84.6)	210 (64.4)	11 (61.1)
Blind/visual loss	0 (0)	U	U	13 (21.3)	0 (0)	107 (32.8)	0 (0)
Redness of eye or eye pain	4 (30.8)	U	U	U	2 (15.6)	46 (14.1)	5 (27.8)
Previous history of syphilis	1 (10.0)	U	15 (38.5)	11 (31.4)	U	U	2 (16.7)
Mean/median duration of symptoms	11.4 ± 16.7 months	U	2.4 weeks	2 months	U	U	U
Mean/median follow up duration	23.1 ± 18.2 months	16.3 months	U	9 months	4.1 ± 5.8 months	U	26 months
Sexual behaviour (%)							
Heterosexual	10 (100)	U	11 (28.2)	U	U	U	10 (83.3)
Bisexual	0 (0)	U	4 (10.3)	U	U	U	2 (16.7)
MSM	0 (0)	U	3 (7.6)	9 (25.7)	0 (0)	249 (68.8)	0 (0)
Unknown	0 (0)	U	21 (53.8)	U	U	U	0 (0)
Laterality (%)							
Both eyes	3 (30.0)	5 (41.6)	6 (15.3)	26 (74.3)	8 (61.5)	U	6 (50)
Unilateral	7 (70.0)	7 (58.3)	33 (84.6)	9 (25.7)	5 (38.4)	U	6 (50)
Visual acuity presentation (%)							
6/12 or better	1 (7.7)	11 (64.7)	U	31 (50.8)	10 (47.6)	U	7 (38.8)
6/15 - 6/60	5 (38.5)	5 (41.7)	U	17 (27.9)	8 (38.1)	U	9 (50.0)
> 6/60	7 (53.8)	1 (5.8)	U	13 (21.3)	1 (4.8)	U	2 (11.1)
Unrecorded	0 (0)	0 (0)	U	0 (0)	2 (9.5)	U	0 (0)
Clinical features (%)							
Anterior uveitis	2 (15.4)	5 (29.4)	3 (6.7)	10 (16.4)	1 (4.8)	NS	6 (33.3)
Intermediate uveitis	0 (0)	2 (11.8)	2 (4.4)	2 (3.3)	0 (0)	NS	1 (5.6)
Anterior uveitis and intermediate uveitis	0 (0)	0 (0)	0 (0)	12 (9.7)	0 (0)	NS	0 (0)
Posterior uveitis	7 (53.8)	5 (29.4)	17 (37.8)	5 (8.2)	13 (61.9)	20 (12.7)	5 (27.8)
Posterior uveitis and intermediate uveitis	0 (0)	0 (0)	3 (6.7)	0 (0)	0 (0)	NS	0 (0)
Panuveitis	4 (30.8)	2 (11.8)	13 (28.9)	28 (45.9)	2 (9.5)	NS	6 (33.3)
Optic nerve involvement	2 (15.4)	1 (5.9)	5 (11.1)	8 (13.6)	5 (23.8)	18 (11.4)	6 (33.3)
Retinal detachment	0 (0)	0 (0)	0 (0)	0 (0)	0 (0)	6 (3.8)	0 (0)
Scleritis	0 (0)	2 (11.8)	0 (0)	4 (6.5)	0 (0)	0 (0)	0 (0)
Treatment (%)							
IV penicilin	9 (90.0)	10 (83.3)	0 (0)	24 (68.6)	0	230 (59.3)	8 (66.7)
IM penicilin	1 (10.0)	0 (0)	14 (35.9)	1 (2.9)	3 (23.0)	NS	4 (33.3)
IV and IM penicilin	0 (0)	0 (0)	10 (25.6)	7 (20.0)	2 (15.4)	NS	0 (0)
Other antibiotics regime	0 (0)	2 (16.7)	15 (38.5)	1 (2.9)	7 (53.8)	NS	0 (0)
No treatment/Unrecorded	0 (0)	0 (0)	0 (0)	2 (5.7)	1 (7.7)	12 (3.1)	0 (0)
Visual acuity final (%)							
6/12 or better	4 (30.8)	13 (76.4)	U	36 (59.0)	1 (4.8)	U	14 (77.8)
6/15 - 6/60	5 (38.5)	4 (23.5)	U	11 (18.0)	11 (52.4)	U	3 (16.7)
> 6/60	4 (30.8)	0 (0)	U	7 (11.4)	1 (4.8)	U	1 (5.6)
Unrecorded	0 (0)	0 (0)	U	7 (11.4)	8 (38.1)	U	0 (0)
Recurrence	2 (20.0)	0 (0)	1 (2.2)	U	U	U	2 (16.7)

Although ocular syphilis can affect almost all structure of the eye, there were differences observed between HIV-negative and HIV-positive patients. Amaratunge et al. reviewed almost 143 descriptions of patients with uveitis caused by syphilis infection. The most important finding of the study was the rate of patients with isolated anterior uveitis, who had a relative risk 14.5 times higher of being diagnosed as HIV-positive as compared to the HIV-negative patients [18]. This high relative risk was also supported by Zhu et al. in China, whose study revealed that none of their 28 HIV-negative patients had isolated anterior uveitis [10]. However, some studies conducted in Europe and South East Asia contradict such results, stating that anterior uveitis was a common presentation in HIV-negative patients [6-7,17]. Our study supports these findings whereby two eyes (14.3%) from our review were diagnosed with isolated anterior uveitis. The discrepancy of the frequency of anterior uveitis can be attributed to the small number of patients enrolled in all mentioned studies, which gave a wide variation in the percentages.

On the other hand, Amaratunge et al. also noted that almost 62.0% of ocular syphilis cases in HIV-negative patients presented with posterior uveitis [18]. This high incidence of posterior uveitis amongst HIV-negative patients mirrors our findings, in which posterior uveitis accounts for 53.8% (seven eyes) of the eyes involved. Other studies of HIV-negative patients have similar findings, with the exception of one study in which higher incidence of posterior and anterior uveitis [5,7,16]. It is interesting to note that if HIV status is not taken into consideration, panuveitis exhibits the most common presentation [4,6].

Ocular syphilis is considered a neurosyphilis variant, meaning the primary treatment for ocular syphilis is administering IV benzylpenicillin for 14 days [19]. This practice adheres to the Malaysian clinical practice guidelines and WHO guidelines for the treatment of neurosyphilis [20-21]. Despite the existence of these guidelines, controversy remains surrounding the classification of isolated anterior uveitis as a neurosyphilis variant. Some clinicians regard isolated anterior uveitis in ocular syphilis as a neurosyphilis risk factor rather than neurosyphilis itself, and therefore its treatment would not follow the neurosyphilis regime. In such cases, IM penicillin injection was used as the only form of treatment, and this yielded satisfactory patient outcomes [17]. In our study, one patient was treated with an IM penicillin injection for isolated anterior uveitis (case 1). However, she returned presenting with recurrent anterior uveitis one year after treatment. Her subsequent treatment then followed the neurosyphilis regime, which showed improvement and no recurrence. This experience, though limited to one incident, influenced us to treat all forms of uveitis in suspected ocular syphilis cases as neurosyphilis infections instead.

In our center, laboratory diagnosis of syphilis followed a reverse syphilis testing algorithm in which patients were investigated with a specific treponemal test (ECLIA, TP-PA, or FTA-ABS), which was subsequently proceeded by quantitative non-treponemal (RPR) testing as confirmation. According to Man Li et al., the reverse syphilis testing algorithm had higher sensitivity and accuracy in detecting syphilis at any stage of infection compared to traditional algorithms (respectively with 99.85% vs. 75.81% sensitivity and 99.96% vs. 97.22% accuracy) [22]. Out of the 10 patients in our review, diagnosis of nine patients (90.0%) adhered to the reverse syphilis testing algorithm. One patient (case 3) exhibited negative syphilis results for both ECLIA and RPR tests during the initial investigation. However, due to clinical suspicion of the syphilis infection, a third laboratory test (FTA-ABS) was conducted, revealing a positive result.

In Table 2, the titer test results of RPR ranged from “non-reactive” to 1/16 titer. Low-to-normal titer results are expected in immunocompetent patients in latent or tertiary stages of the syphilis infection [7]. A problem arose in case 2 when the RPR level was negative while the ECLIA test exhibited positive results. This gave the impression of a false reactivity. When a case exhibits such discordance between ECLIA and RPR testing, a third, confirmatory laboratory test is mandatory [7]. Therefore, the patient was subsequently tested with TP-PA for confirmatory diagnosis and recorded as positive.

The improvement of visual acuity post-IV benzylpenicillin treatment can be seen in all types of uveitis. General improvement in visual acuity was seen in 11 eyes (84.6%). Patients with isolated posterior uveitis (five eyes) and posterior uveitis with optic neuritis (two eyes) were observed to have poorer visual acuity during the presentation (worse than 6/60) when compared with visual acuity results of other types of uveitis. Out of seven eyes with posterior uveitis, four (57.1%) exhibited vision improvement quantified by more than three Snellen lines. This improvement shows the importance of diagnosing and treating ocular syphilis early. Appropriate and timely treatment is vital in anticipating good visual outcomes [6].

This study has one important limitation. Due to the rare occurrence of the diseases, only a limited number of ocular syphilis cases were studied, which may not be enough for proper statistical analysis. This situation occurred in other similar studies as well. However, the contribution of this small amount of data may assist in future reviews of this disease, thus contributing to an accurate analysis of the presentation and outcomes of ocular syphilis. For future research, a multicentre study should also be conducted to accurately assess the prevalence of ocular syphilis.

## Conclusions

Diagnosis of ocular syphilis poses challenges as a result of its rarity and its diverse manifestations. A high index of suspicion with early diagnosis and treatment are crucial in achieving good visual outcomes and low recurrence rates. Sequential follow-up is also recommended because recurrence or relapse can occur despite completing treatments.
